# Diversity of the causal genes in hearing impaired Algerian individuals identified by whole exome sequencing

**DOI:** 10.1002/mgg3.131

**Published:** 2015-02-15

**Authors:** Fatima Ammar-Khodja, Crystel Bonnet, Malika Dahmani, Sofiane Ouhab, Gaelle M Lefèvre, Hassina Ibrahim, Jean-Pierre Hardelin, Dominique Weil, Malek Louha, Christine Petit

**Affiliations:** 1 Equipe de Génétique, Laboratoire de Biologie Moléculaire, Faculté des Sciences Biologiques, Université des Sciences et de la Technologie Houari Boumédiène (USTHB)Alger, Algeria; 2 Institut de la Vision, UMRS 1120 INSERM/UPMC/Institut PasteurParis, France; 3 Service d'Otorhinolaryngologie (ORL), Hôpital de Kouba-Bachir MentouriAlger, Algeria; 4 Service d'Otorhinolaryngologie (ORL), Hôpital Mustapha PachaAlger, Algeria; 5 Institut Pasteur, Unité de Génétique et Physiologie de l'AuditionUMRS 1120 INSERM/UPMC Paris 6, Paris, France; 6 Service de Biochimie, Hôpital Armand TrousseauUMRS 1120 INSERM, Paris, France; 7 Collège de FranceParis, France

**Keywords:** Algeria, deafness, genetic heterogeneity, whole exome sequencing

## Abstract

The genetic heterogeneity of congenital hearing disorders makes molecular diagnosis expensive and time-consuming using conventional techniques such as Sanger sequencing of DNA. In order to design an appropriate strategy of molecular diagnosis in the Algerian population, we explored the diversity of the involved mutations by studying 65 families affected by autosomal recessive forms of nonsyndromic hearing impairment (DFNB forms), which are the most prevalent early onset forms. We first carried out a systematic screening for mutations in *GJB2* and the recurrent p.(Arg34*) mutation in *TMC1*, which were found in 31 (47.7%) families and 1 (1.5%) family, respectively. We then performed whole exome sequencing in nine of the remaining families, and identified the causative mutations in all the patients analyzed, either in the homozygous state (eight families) or in the compound heterozygous state (one family): (c.709C>T: p.(Arg237*)) and (c.2122C>T: p.(Arg708*)) in *OTOF*, (c.1334T>G: p.(Leu445Trp)) in *SLC26A4*, (c.764T>A: p.(Met255Lys)) in *GIPC3*, (c.518T>A: p.(Cys173Ser)) in *LHFPL5*, (c.5336T>C: p.(Leu1779Pro)) in *MYO15A*, (c.1807G>T: p.(Val603Phe)) in *OTOA*, (c.6080dup: p.(Asn2027Lys*9)) in *PTPRQ*, and (c.6017del: p.(Gly2006Alafs*13); c.7188_7189ins14: p.(Val2397Leufs*2)) in *GPR98*. Notably, 7 of these 10 mutations affecting 8 different genes had not been reported previously. These results highlight for the first time the genetic heterogeneity of the early onset forms of nonsyndromic deafness in Algerian families.

## Introduction

Approximately 1 in 700 children is affected by severe or profound hearing impairment at birth or during early childhood (prelingual deafness) (Morton 1991). Most cases are attributable to a genetic cause, typically monogenic. About 30% of the genetic cases are syndromic, that is, deafness is associated with other clinical anomalies, whereas 70% are nonsyndromic or isolated, that is, the auditory defect is the sole clinical manifestation (Holder 1996). The inheritance modes of isolated deafness can be autosomal dominant, autosomal recessive, X or Y chromosome-linked, or mitochondrial. Identification of the causative mutations in affected individuals can be difficult due to the high degree of genetic heterogeneity. Indeed, 77 different genes have already been identified for DFNB (deafness autosomal recessive) and DFNA (deafness autosomal dominant) forms (Hereditary Hearing Loss Homepage: http://hereditaryhearingloss.org/).

About 85% of disease-related mutations in Mendelian disorders have been found in the protein-coding regions of genes (exons and splice sites), which only represent about 1% of the human genome (Teer and Mullikin 2010). Molecular diagnosis of autosomal recessive nonsyndromic hearing impairment consists in screening many, and sometimes, long exons for mutations, making conventional methods (e.g., Sanger sequencing) expensive and time-consuming (Diaz-Horta et al. 2012). Advances in next-generation sequencing technologies, however, have made possible to sequence virtually all exons at a time (the so-called “whole exome sequencing”), and therefore to rapidly identify mutations responsible for Mendelian disorders. Whole exome sequencing has thus become an efficient and cost-effective alternative approach for molecular diagnosis (Choi et al. 2009; Ng et al. 2010), and has successfully been used to identify new causative genes or new mutations in genes involved in syndromic and nonsyndromic forms of hearing impairment (Delmaghani et al. 2012; Diaz-Horta et al. 2012; de Keulenaer et al. 2012; Wei et al. 2012; Bonnet et al. 2013).

Mutations in *GJB2* (OMIM*121011), encoding connexin 26 at the *DFNB1* locus, are highly prevalent in Maghrebian populations, accounting for approximately 40% of the DFNB cases in Algeria (Ammar-Khodja et al. 2009), 35% in Tunisia (Riahi et al. 2013), and 37% in Morocco (Abidi et al. 2007). In addition, the p.(Arg34*) nonsense mutation in *TMC1* (OMIM*606706), encoding transmembrane channel-like 1 at the DFNB7/11 locus, would account for 3–5% of DFNB cases in Algeria and Tunisia (Ben Said et al. 2010). However, the extent of genetic heterogeneity of prelingual hearing impairment in Maghrebian countries remains to be determined. Here, our objective was to assess the diversity of the genes involved in the Algerian population. This is particularly relevant to such a highly consanguineous population in the perspective of developing molecular diagnosis.

## Patients and Methods

### Patients

Patients were recruited and clinically examined in otorhinolaryngology centers and deafness schools in Alger, Algeria. Patients with a known environmental cause of hearing impairment, including infection, premature delivery, head trauma, and use of ototoxic drugs, were not included in the study. Hearing levels were measured by pure tone audiometry, which included air and bone conduction. Hearing thresholds were obtained for sound frequencies between 250 and 8000 Hz. All patients exhibited bilateral, moderate (45–65 dB) to severe (70–89 dB), or profound (>90 dB) sensorineural hearing loss. In all the families, the parents of the affected siblings were normal-hearing. Clinical examination of the patients did not give indication for a syndromic deafness. Ocular fundus examination was normal, and neither proteinuria nor hematuria was detected. The temporal bone CT scan analysis could not be carried out in all the patients, but did not show cochleovestibular malformations in the patients analyzed. The vestibular function was not tested clinically, but a questionnaire on the early sitting and walking steps was filled in. This study was approved by the local ethics committees, and consent to genetic testing was obtained from adult probands or from the parents when the patient was under 18 years.

### DNA extraction and sequencing

Genomic DNA was extracted from peripheral blood lymphocytes using the Promega Wizard Genomic DNA Purification Kit (Promega, Madison, MI) (Cat. #A1120) according to the manufacturer's instructions. Screening for mutations in *GJB2* and in exon 7 of *TMC1* was performed by Sanger sequencing. Whole exome sequencing and bioinformatic analysis were carried out on pooled DNA samples from two affected siblings in each family, as previously described (Delmaghani et al. 2012). The Sanger sequencing technique was then used on individual exons to validate each pathogenic mutation identified by whole exome sequencing, and to confirm its presence in the homozygous state in all affected siblings, and in the heterozygous state in the parents. To amplify and sequence exons, specific oligonucleotides were designed using Primer3 (http:/frodo.wi.mit.edu/primer3/) (Table S1). PCR amplification and Sanger sequencing were carried out as previously described (Delmaghani et al. 2012). GenBank reference sequences of each genes studied are mentioned in Table1.

**Table 1:** Mutations found in Algerian DFNB families.

Family	Gene	Genotype	Amino acid change	Phenotype
1	*NM_194248.2* *(OTOF)*	c.699C>T/c.699C>T	p.(Arg237^*^)	Profound deafness/auditory neuropathy
2	*NM_194248.2* *(OTOF)*	c.2122C>T/c.2122C>T	p.(Arg708^*^)	Profound deafness
3	*NM_000441.1* *(SLC26A4)*	c.1334T>G/c.1334T>G	p.(Leu445Trp)	Profound deafness + enlarged vestibular aqueduct
4	*NM_133261.* *(GIPC3)*	**c.764T>A/c.764T>A**	**p.(Met255Lys)**	Profound deafness
5	*NM_182548.3* *(LHFPL5)*	**c.518T>A/c.518T>A**	**p.(Cys173Ser)**	Profound deafness
6	*NM_016239.3* *(MYO15A)*	**c.5336T>C/c.5336T>C**	**p.(Leu1779Pro)**	Profound deafness
7	*NM_144672.3* *(OTOA)*	**c.1837G>T/c.1837G>T**	**p.(Val603Phe)**	Severe-to-profound deafness
8	*NM_001145026.1* *(PTPRQ)*	**c.5592dup/c.5592dup**	p.(Glu134Glyfs^*^6)	Profound deafness
9	*NM_032119.3* *(GPR98)*	**c.6017del/c.7188_7189ins14**	p.(Gly2006Alafs^*^13)/p.(Val2397Leufs^*^2)	Moderate deafness + retinal defect (Usher syndrome of type II)

Novel mutations are indicated in bold.

## Results and Discussion

Sixty-five Algerian families, comprised two to four siblings affected with bilateral, moderate (45–69 dB), severe (70–89 dB), or profound (>90 dB) autosomal recessive prelingual hearing impairment, were studied. In 80% of these families, that is, 52 families, hearing impaired children were born to consanguineous parents.

The first step in molecular diagnosis consisted in the screening of the *GJB2* single coding exon, and of the p.(Arg34*) mutation in *TMC1* by Sanger sequencing. Mutations in *GJB2* accounted for 47.7% of the cases (31 of 65 patients). The c.35delG: p.(Gly12Valfs*2) mutation was present in the homozygous state in 25 (80%) of 31 patients, and in the compound heterozygous state (c.35delG: p.(Gly12Valfs*2)/c.139G>T: p.(Glu47*)) in 5 (16%) patients, whereas 1 (3.2%) patient carried the c.139G>T: p.(Glu47*) mutation in the homozygous state. The c.100C>T: p.(Arg34*) nonsense mutation in *TMC1* was identified in one patient, in the homozygous state.

**Figure 1:**
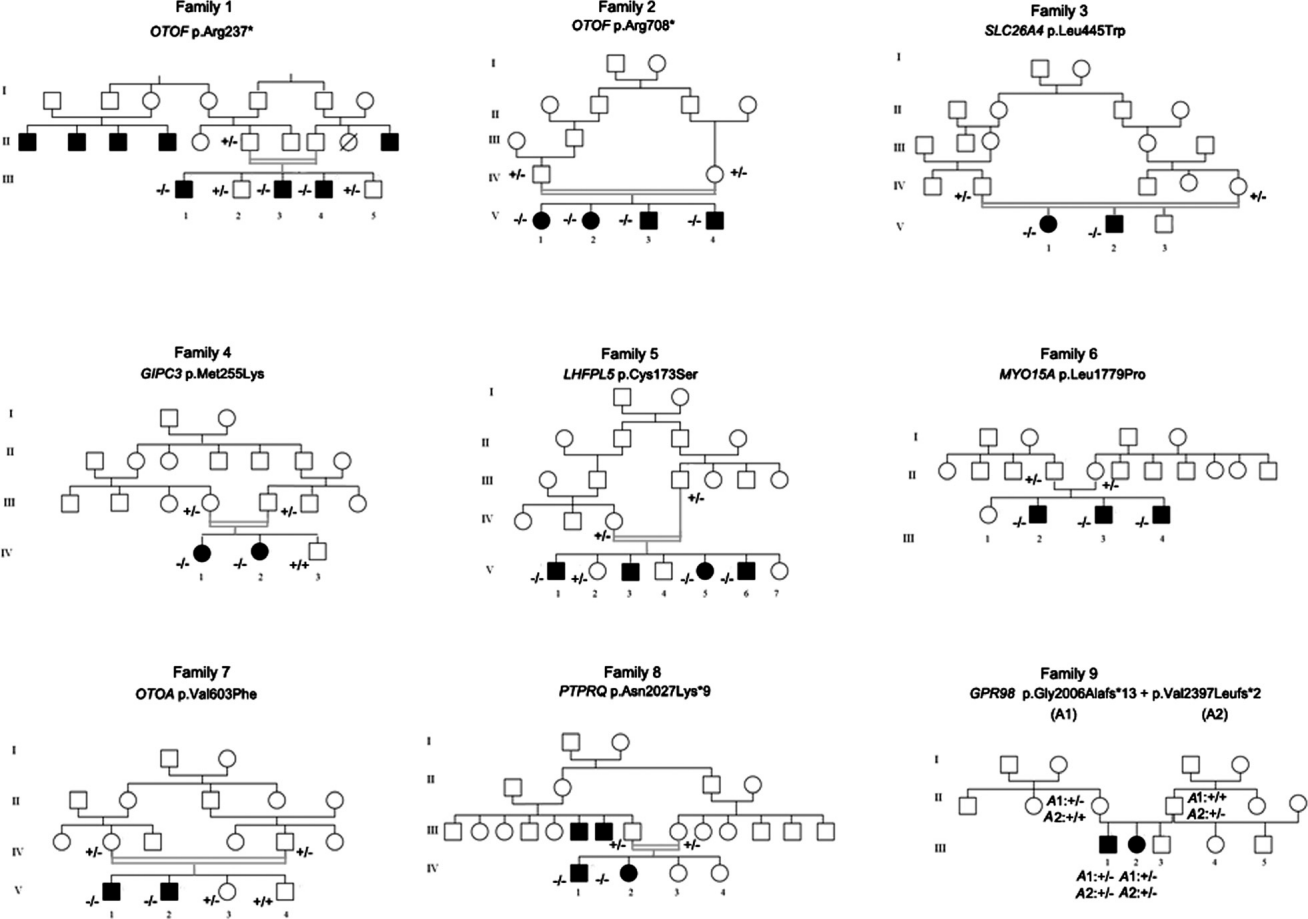
Pedigree of the nine Algerian families.

Nine unrelated pairs of affected siblings were selected from the 33 remaining families (based on several criteria including different geographic origins, large sets of siblings, and parental consanguinity), and underwent whole exome sequencing analysis. The pedigrees are shown in Figure1. To analyze the data, we first excluded sequence variants with prevalence higher than 3% in the dbSNP132, 1000 genomes, and HapMap databases. Second, we focused on variants present in the coding exons and flanking splice sites (nonsense, frameshift, missense, or splice site mutations). Finally, in the seven pairs of patients born to consanguineous parents, we expected the causative mutations to be present in the homozygous state. In all nine families, we identified presumably pathogenic biallelic mutations in known deafness genes. These mutations are listed in Table1, together with the patients’ auditory phenotypes. Among the 10 different mutations identified, seven had not been reported previously and were not present in 200 Algerian control alleles or in the 1000 genomes and Exome Variant Server databases.

Nonsense mutations in *OTOF* (DFNB9, OMIM*603681) (Yasunaga et al. 1999), c.699C>T: p.(Arg237*) and c.2122C>T: p.(Arg708*), each in the homozygous state, were found in patients III.1, III.3, III.4, and V.1, V.2, V.3, V.4 from families 1 and 2, respectively. These mutations had previously been reported in two families from the United Arab Emirates (Houseman et al. 2001) and from Spain. Of note, patients III.1, III.3, and III.4 presented with auditory neuropathy. *OTOF* encodes otoferlin, a large transmembrane protein involved in exocytosis of synaptic vesicles at the inner hair cell ribbon synapse (Roux et al. 2006). Mutations in *OTOF* usually result in prelingual, profound deafness (Chaib et al. 1996; Yasunaga et al. 2000). To date, more than 90 pathogenic sequence variants in *OTOF* have been reported (Mahdieh et al. 2012). The p.(Gln829*) nonsense mutation is responsible for about 3% of all DFNB cases in Spain (Migliosi et al. 2002; Rodriguez-Ballesteros et al. 2003), but this mutation was not detected in our patients.

In patients V.1 and V.2 from family 3, we identified a biallelic missense mutation (c.1334T>G: p.(Leu445Trp)) in exon 11 of *SLC26A4* (DFNB4, OMIM*605646). This mutation had been previously reported in one Dutch family and two Tunisian families (van Hauwe et al. 1998; Masmoudi et al. 2000). The mutation is predicted to be pathogenic by PolyPhen-2, SIFT, and Mutation Taster (Table S2), and has indeed been shown to prevent the targeting of the protein to the plasma membrane in transfected COS-7 cells (Choi et al. 2009). In the patients from family 3, temporal bone CT scan analysis was carried out after the genetic analysis and showed bilateral enlargement of the vestibular aqueduct, a common inner ear malformation in DFNB4 patients (Usami et al. 1999). *SLC26A4* encodes pendrin, a transmembrane ion transporter exchanging chloride for other anions, such as iodide in the thyroid gland or bicarbonate in the inner ear. In the cochlea, pendrin is found in the apical membrane of outer sulcus and spiral prominence epithelial cells and in supporting cells, which border the endolymphatic compartment, and in cochlear ganglion cells (Yoshino et al. 2006). Mutations in *SLC26A4* are responsible for both syndromic (Pendred syndrome) and nonsyndromic (DFNB4) hearing impairment (Li et al. 1998; Blons et al. 2004). Mutations in this gene have been reported to account for as many as 7–13% of all deafness cases in Chinese and Danish populations (Yuan et al. 2012; Rendtorff et al. 2013).

Patients IV.1 and IV.2 in family 4 carried a previously unreported biallelic missense mutation in *GIPC3* (OMIM*608792, c.764T>A: p.(Met255Lys), RNA not analyzed), which was absent in their unaffected brother. This mutation is predicted to be pathogenic by two of the three algorithms used (Table S2). *GIPC3* (G alpha Interacting Protein C terminus 3), the gene responsible for DFNB15/95/72 (Ain et al. 2007), encodes a PDZ-domain-containing protein involved in the postnatal maturation of the hair bundles and the long-term survival of inner ear hair cells and cochlear ganglion cells (Charizopoulou et al. 2011). To date, 10 pathogenic sequence variants in *GIPC3* have been reported (Masaru 2013). Different variants are present in India (Charizopoulou et al. 2011), Pakistan (Rehman et al. 2011), Turkey (Sirmaci et al. 2009; Diaz-Horta et al. 2012), and Saudi Arabia (Ramzan et al. 2013). These mutations cause variable hearing impairment, from moderate to profound. In family 4, patients IV.1 and IV.2 both presented with bilateral, severe-to-profound prelingual deafness, as did the patients carrying a previously reported missense mutation affecting the next amino acid residue within the GH2 domain of the protein (c.767G>A: p.(Gly256Asp)) (Masaru 2013).

Patients V.1, V.5, and V.6 in family 5 carried a previously unreported biallelic missense mutation in *LHFPL5* (OMIM*609427, c.518 T>A: p.(Cys173Ser), RNA not analyzed). In patient V.3, DNA was not available to identify the mutation. This mutation is predicted to be pathogenic by the three algorithms used (Table S2). *LHFPL5* (lipoma HMGIC fusion partner-like 5), the gene responsible for DFNB67 (Kalay et al. 2006) encodes TMHS (tetraspan membrane protein of hair cell stereocilia). This protein is present in the stereocilia of inner and outer hair cells of the cochlea (Longo-Guess et al. 2005), and is involved in the auditory mechano-electrical transduction (Xiong et al. 2012). The mutation affects a cysteine residue in the second extracellular loop of the protein (between transmembrane domains 3 and 4), and points to an essential role of this residue in the structure and/or the function of the protein.

Patient III.2, III.3, and III.4 in family 6 carried a previously unreported biallelic missense mutation in *MYO15A* (OMIM*602666, c.5336A>G: p.(Leu1779Pro), RNA not analyzed). This mutation, the first *MYO15A* mutation identified in Algeria, is predicted to be pathogenic by the three algorithms used (Table S2). *MYO15A* is the gene responsible for DFNB3 (Fridman et al. 1995). It encodes myosin XVa, a large actin-based motor protein of cochlear hair cells. The mutation affects an amino acid residue located in the motor domain of the protein, and is therefore predicted to be deleterious for its motor activity. In the cochlear hair cells, myosin XVa plays an important role in the differentiation and elongation of the stereocilia (Belyantseva et al. 2003). Some mutations in *MYO15A* have been reported in populations from Tunisia, Pakistan, India, Turkey, Indonesia, and Brazil (Belguith et al. 2009; Cengiz et al. 2010; Bashir et al. 2012; Fattahi et al. 2012; Riahi et al. 2014).

Patients V.1 and V.2 in family 7 carried a previously unreported biallelic mutation in *OTOA* (OMIM*607039, c.1807G>T: p.(Val603Phe), RNA not analyzed). This missense mutation is predicted to be pathogenic by the three algorithms used, and may also interfere with the acceptor site of intron 18 according to the NNSPLICE prediction program (Table S2). *OTOA*, responsible for DFNB22, encodes otoancorin, an inner ear-specific glycosylphosphatidylinositol-anchored protein present at the apical surface of spiral limbus cells in the cochlea (Zwaenepoel et al. 2002). This protein is required for limbal attachment of the tectorial membrane, which conditions the proper stimulation of the inner hair cells, the genuine auditory sensory cells (Lukashkin et al. 2012). No mutations in *OTOA* have so far been reported in Tunisia and Morocco.

Patients IV.1 and IV.2 in family 8 carried an unreported biallelic frameshift duplication in *PTPRQ* (OMIM*603317, c.6080dup: p.(Asn2027Lys*9), RNA not analyzed) introducing a premature stop codon. *PTPRQ* (protein tyrosine phosphatase, receptor type, Q), responsible for DFNB84 (Shahin et al. 2010), encodes a member of the type III receptor-like protein tyrosine phosphatase family. Only three mutations (two nonsense and a missense) have previously been reported in *PTPRQ*, in Dutch, Moroccan, and Palestinian families (Schraders et al. 2010; Shahin et al. 2010). Dutch and Moroccan patients also had abnormal vestibular function (Schraders et al. 2010). Patients IV.1 and IV.2 in family 8 have profound deafness without delay in walking age, suggesting the absence of vestibular dysfunction.

Finally, patients III.1 and III.2 in family 9 carried two different frameshift mutations in *GPR98*, (OMIM*602851, c.6017del: p.(Gly2006Alafs*13), RNA not analyzed) and (c.7188_7189ins14: p.(Val2397Leufs*2), RNA not analyzed). Segregation analysis confirmed the biallelic inheritance of the mutations from the parents (Fig.1). *GPR98*, encoding the G protein-coupled receptor 98, also known as VLGR1 (very large G protein-coupled receptor 1) is responsible for Usher syndrome of type 2C, characterized by bilateral, mild-to-moderate sensorineural hearing impairment, normal vestibular function, and progressive-onset visual loss associated with retinitis pigmentosa (Weston et al. 2004). Consistently, the two patients of family 9 had moderate deafness and, although the initial ocular fundus examination did not reveal any abnormalities, further ophthalmological evaluation by electroretinogram confirmed the molecular diagnosis of Usher syndrome by showing abnormal photoreceptor function in all retinal regions.

In conclusion, our study, the first one analyzing a large number of families affected by early onset nonsyndromic hearing impairment in Algeria, not only shows that mutations in *GJB2* account for a large proportion of the Algerian DFNB cases (48% of the families analyzed) but also reveals a substantial heterogeneity in the causal genes, with 7 of 10 mutations detected in eight different genes being not previously reported. Of note, the three previously reported mutations had been found in DFNB families from Saudi Arabia, Tunisia, and Spain, therefore opening the possibility to trace population migrations through the search of these mutations in other countries around the Mediterranean Sea and in the Middle East.

## Supporting Information

**Table S1.** Primers used to validate the mutations by Sanger sequencing.

**Table S2.** Pathogenicity predictions of missense mutations.
