# Supplementary material for: Diversity of the causal genes in hearing impaired Algerian individuals identified by whole exome sequencing
Source: Mol Genet Genomic Med. 2015 Feb 15;3(3):189–96. doi: 10.1002/mgg3.131 (PMC4444160; doi:10.1002/mgg3.131)
Supplement: Supplementary file 1 — Table S1. Primers used to validate the mutations by Sanger sequencing. [file mgg30003-0189-sd1.docx]

**Suppl. Table 1**: Primers used to validate the mutations by Sanger sequencing

| **Gene** | **Exon** | **Forward primer (5'-3')** | **Reverse primer (5'-3')** |
| --- | --- | --- | --- |
| ***GIPC3*** | Exon 5 | GCCTCCCAGGGTTTACAAAG | CCTCCTCCCGTAGGCTTC |
| ***LHFPL5*** | Exon 2 | CATGTGCACCCCTCCTTC | CCATTGTGGAGTTGGGTGAT |
| ***MYO15A*** | Exon 20 | GACCCTGCCTGTCTGTTTTC | GGTCATGAGGTGGGTTAAGG |
| ***PTPRQ*** | Exon 35 | AAGTGAAATTTCTTGTTTACCTCTGA | GGTAGGCATTTAAAAATATTTCCTAGC |
| ***OTOA*** | Exon 19 | TGACTGGCAAAGAATGTTACTCA | TCGAGGAGGGTTCTATGTGC |
| ***OTOF*** | Exon 7  Exon 18 | CAGCCCACCTAACCAGTCTTTCA  CAGCCTCCCATCCTCCTGT | CCGTCCATGAGCCCTGATTCT  CTGGGCAGACCAGCTTTGT |
| ***SLC26A*** | Exon 11 | GACACAAGGGAGAAGGACGA | GGTTTTCCATGTGATGATTGTT |
| ***GPR98*** | Exon 28  Exon 33 | GCCTCTAAGTAACTGGCTTTAATC  AAGACCACAGTCAGCATTCCTC | TGTTTCAGGATTCTGAGAGCTAA  TCATGTCAACCATTAACAAGCC |
