# Supplementary material for: Diversity of the causal genes in hearing impaired Algerian individuals identified by whole exome sequencing
Source: Mol Genet Genomic Med. 2015 Feb 15;3(3):189–96. doi: 10.1002/mgg3.131 (PMC4444160; doi:10.1002/mgg3.131)
Supplement: Supplementary file 2 — Table S2. Pathogenicity predictions of missense mutations. [file mgg30003-0189-sd2.docx]

**Suppl. Table 2:** Pathogenicity predictions of missense mutations

| **Mutation** | **PolyPhen-2 (score)** | **SIFT (score)** | **Mutation Taster (score)** | **NNSPLICE** |
| --- | --- | --- | --- | --- |
| Met255Lys  (*GIPC3*) | Benign (0.13) | Deleterious (0) | Disease causing (1) | - |
| Cys173Ser  (*LHFPL5*) | Probably damaging (1.00) | Deleterious (0) | Disease causing (1) | - |
| Leu1779Pro  (*MYO15A*) | Probably damaging (1.00) | Deleterious (0) | Disease causing (1) | - |
| Val603Phe  (*OTOA*) | Probably damaging (0.97) | Deleterious (0.02) | Disease causing (1) | Potential splice site interference (-26%) |
| Leu445Trp  (*SLC26A4*) | Probably damaging (1.00) | Deleterious (0) | Disease causing (1) |  |
